# Temperature modulates the osmosensitivity of tilapia prolactin cells

**DOI:** 10.21203/rs.3.rs-2524830/v1

**Published:** 2023-02-28

**Authors:** Tharindu Malintha Gardi Hewage, Daniel W. Woo, Fritzle T. Celino-Brady, Andre P. Seale

**Affiliations:** University of Hawai’i at Mānoa; University of Hawai’i at Mānoa; University of Hawai’i at Mānoa; University of Hawai’i at Mānoa

## Abstract

In euryhaline fish, prolactin (Prl) plays an essential role in freshwater (FW) acclimation. In the euryhaline and eurythermal Mozambique tilapia, *Oreochromis mossambicus*, Prl cells are model osmoreceptors, recently described to be thermosensitive. To investigate the effects of temperature on osmoreception, we incubated Prl cells of tilapia acclimated to either FW or seawater (SW) in different temperature (20, 26 and 32°C) and osmolality (280, 330 and 420 mOsm/kg) combinations for 6 h. Release of both Prl isoforms, Prl_188_ and Prl_177_, increased in hyposmotic media and were further augmented with a rise in temperature. Hyposmotically-induced release of Prl_188_ was inhibited at 20°C. In SW fish, mRNA expression of *prl_188_* and *prl_177_* showed direct and inverse relationships with temperature, respectively. In SW-acclimated tilapia Prl cells incubated in hyperosmotic media, Prl receptors, *prlr1* and *prlr2*, and the stretch-activated Ca^2+^ channel, *trpv4*, were inhibited at 32°C, suggesting the presence of a cellular mechanism to compensate for elevated Prl release. Transcription factors, *pou1f1*, *pou2f1b*, *creb3l1*, *cebpb*, *stat3*, *stat1a* and *nfat1c*, known to regulate *prl_188_* and *prl_177_*, were also downregulated at 32°C. Our findings provide evidence that osmoreception is modulated by temperature, and that both thermal and osmotic responses vary with acclimation salinity.

## Introduction

In vertebrates, hydromineral balance is maintained through osmoregulation. Osmoregulatory processes, in turn, are largely mediated through osmosensitive cells and the neuroendocrine system ^1,2^. Euryhaline fishes, which are characterized by their capacity to thrive in a wide range of environmental salinities, have been employed to elucidate the mechanisms underlying the transduction of osmotic stimuli^3-7^. More recently, in light of impending climate-change driven changes in environmental temperature and salinity, a need for cellular and organismal models where the integration of distinct thermal and osmotic stimuli can be studied has emerged ^8,9^.

Prolactin (Prl) is a pleiotropic hormone that exerts hundreds of physiological functions in vertebrates including lactation, osmoregulation, growth, reproduction and immune function ^10-12^. In euryhaline fish, the main function of Prl is to stimulate ion absorption and retention in osmoregulatory tissues to maintain osmotic balance in fresh water (FW)^13,14^. Mozambique tilapia (*Oreochromis mossambicus*) has been widely used to study the effects of Prl on osmoregulation due to its euryhalinity and the morphology of Prl secreting cells, which comprise a nearly homogeneous portion of the *rostral pars distalis* (RPD) of the pituitary ^15,16^. Consistent with its role in FW adaptation, plasma Prl levels are high in FW and its release increases in pituitaries and dispersed Prl cells incubated in hyposmotic media ^17-19^. Tilapia Prl cells secrete two isoforms of Prl, Prl_188_ and Prl_177_, which are encoded by separate genes ^20,21^. Both Prl isoforms act through Prl receptors, Prlr1 and Prlr2, which have been shown to exert distinct downstream effects through JAK/STAT activation and differentially respond to changes in extracellular osmolality ^17,22^. Prl_188_ responds more robustly to hyposmotic stimuli than Prl_177_^17,23^. Due to their importance in FW adaptation, both *prl_188_* and *prl_177_* are found to be 10–30 times higher in Prl cells in FW-acclimated tilapia compared with their seawater (SW) counterparts ^24,25^. Consequently, hyposmotically-induced *prl* expression is more responsive in tilapia acclimated to SW than those in FW^17,26^. Recently, we reported that tilapia Prl cells are also thermosensitive ^27^. Insamuch as Mozambique tilapia is both euryhaline and eurythermal, surviving in salinities ranging from FW to over double-strength SW and temperatures between 10–38°C ^28^, it is likely that both thermal and osmotic stimuli interact during adaptive hormonal responses.

The cellular mechanisms underlying osmoreception in tilapia Prl cells have been recently reviewed ^29^. Briefly, when extracellular osmolality drops, water enters the Prl cell through aquaporin 3 channels (Aqp3) leading to an increase in cell volume and activation of the stretch-activated ion channel, transient receptor potential vanilloid 4 (Trpv4), which enables extracellular Ca^2+^ into the cell^30-34^. An increase in intracellular [Ca^2+^] alone, or through the activation of the cyclic AMP (cAMP) secondary messenger system, increases Prl release^30,35^. Prl also exerts autocrine responses on Prl cells which are in turn modulated by extracellular osmolality^36^. Thermally-induced Prl release also appears to operate through a cell-volume dependent mechanism ^27^. The mechanistic commonalities between hyposmotically- and thermally-induced release of Prls, raise the question of whether similar mechanisms are present in the regulation of *prl* genes.

Recently, several putative transcription factors (TF) predicted to bind promoter regions of *prl_177_* and *prl_188_* were identified ^29^ and their activities measured in the tilapia Prl cell model^37^. Among them, several POU family TFs, such as pituitary transcription factor 1 (Pit1, also known as Pou1f1), a key regulator of pituitary cell differentiation ^38^, were directly responsive to changes in extracellular osmolality^37^. Pit1 shares a common binding site on the tilapia *prl_188_* promoter region with Octamer 1 (Oct1, also known as Pou2f1), another POU family TF ^29^ activated by stressors^39-41^. The roles of cAMP and Ca^2+^ second messenger systems in cellular signaling have been studied, including downstream activation of CAAT/enhancer binding protein (CEBP) and cAMP response element binding protein (CREB) ^42,43^, two TFs also predicted to bind *prl_188_* and *prl_177_* promoter regions ^29^ and recently shown to be hyposmotically induced in tilapia Prl cells^37^. On the other hand, the nuclear factor of activated T cells (NFAT) is hyperosmosensitive, leading to the production of secondary metabolites ^44,45^; binding sites for NFAT were found in the promoter region of tilapia *prl_177_*, but not *prl_188_* ^29^. It remains unclear how thermal stimuli may modulate osmosensitive TFs to regulate *prl* genes.

Previous studies have shown that low temperatures (15°C) reduces the salinity tolerance of Mozambique tilapia and its hybrids ^46,47^. The expression of *trpv4*, is elevated by temperature in chum salmon (*Oncorhynchus keta*) ^48^ and Mozambique tilapia Prl cells ^27^, further reinforcing the crosstalk between thermal and osmotic stimuli in the regulation of Prl synthesis and release. While the tilapia Prl cell model has allowed for the identification of several downstream components involved in the transduction of hyposmotic stimuli into Prl secretion, little is known on how temperature interacts with extracellular osmolality in the regulation of *prl* transcription. Specifically, the identification of common molecular mechanisms of *prl* transcription in response to both thermal and osmotic stimuli shall shed light into how Prl cells and other endocrine systems may integrate environmental stimuli with adaptive physiological responses.

In the present study, we employed dispersed Prl cells from SW- and FW- acclimated tilapia in static incubation experiments to investigate Prl_188_ and Prl_177_ release and transcriptional responses of, *prl_188_, prl_177_, prlr1, prlr2, pou1f1, pou2f1b, creb3l1, cebpb, stat3, stat1a*, and *nfatc1* to changes in osmolality and temperature. This experimental approach allows for the assessment of complex interactions between a fundamental sensory modality, osmoreception, and thermal sensitivity in the endocrine response of a teleost fish model.

## Results

### 1. Effects of temperature and osmolality on Prl release

**Figure 1:**
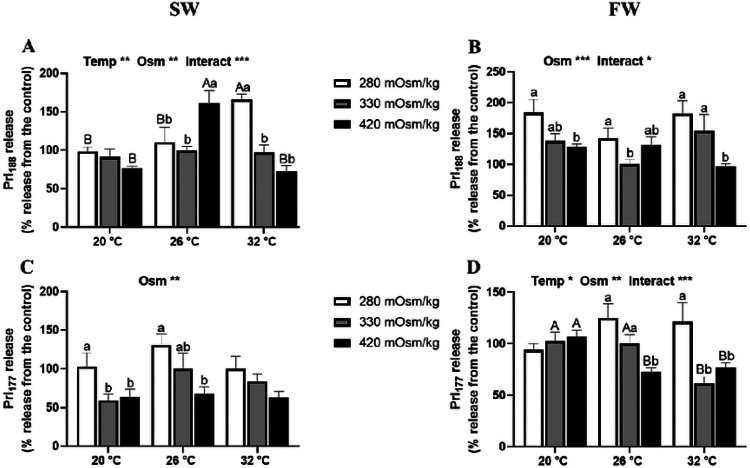
Effects of incubation osmolality and temperature on Prl_188_ and Prl_177_ release from Prl cells of SW-acclimated Mozambique tilapia (A and C) and FW-acclimated tilapia (B and D) following 1 h of incubation. Data are expressed as mean percentage release from the isosmotic and isothermal (330 mOsm/kg:26 °C) group ± SEM (n=6-8). The effects of osmolality and temperature were analyzed by two-way ANOVA (*P<0.05, **P<0.01, ***P<0.001). When there was a significant effect of temperature (Temp), media osmolality (Osm) or interaction (Interact), group comparisons were conducted using protected Fisher’s LSD test. Groups not sharing uppercase letters indicate significant (*P*<0.05) mean differences in response to incubation temperatures and groups not sharing lowercase letters reflect significant (*P*<0.05) mean differences in response to media osmolality.

The effects of temperature and osmolality on Prl_188_ and Prl_177_ released from Prl cell incubations of tilapia acclimated to FW and SW by 1 h are shown in Fig. 1. In Prl cells of SW-acclimated tilapia, effects of both osmolality and temperature were seen in Prl_188_ release by 1 h (Fig. 1A). By 1 h, hyposmotically-induced Prl_188_ release was only observed at 32°C. In FW-acclimated tilapia Prl cells, only an osmotic effect was seen by 1 h (Fig. 1B), with hyposmotically-induced Prl_188_ release observed at all incubation temperatures. In SW fish, only osmolality had an effect on Prl_177_ release by 1 h (Fig. 1C); Prl_177_ release was inversely related to extracellular osmolaity at 20 and 26°C. By contrast, Prl_177_ release from FW-tilapia Prl cells showed both thermal and osmotic effects (Fig. 1D). Hyposmotically-induced Prl release was seen at both 20°C and 26°C, while a rise in temperature inhibited Prl_177_ release in isosmotic and hyperosmotic conditions.

**Figure 2:**
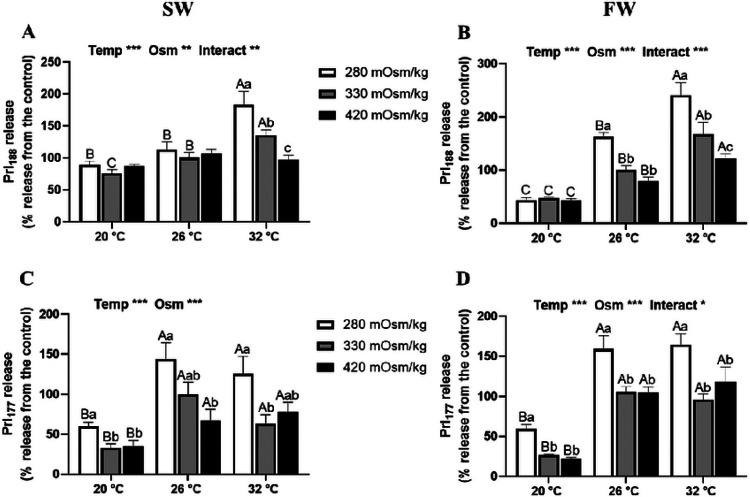
Effects of incubation osmolality and temperature on Prl_n88_ and Prl_177_ release from Prl cells of SW-acclimated Mozambique tilapia (A and C) and FW-acclimated tilapia (B and D) at the end of 6 h incubation. Data are expressed as mean percentage release from the isosmotic and isothermal (330 mOsm/kg:26 °C) group ± SEM (n=6-8). The effects of osmolality and temperature were analyzed by two-way ANOVA (*P<0.05, **P<0.01, ***P<0.001). When there was a significant effect of temperature (Temp), media osmolality (Osm) or interaction (Interact), group comparisons were conducted using protected Fisher’s LSD test. Groups not sharing uppercase letters indicate significant (*P*<0.05) mean differences in response to incubation temperatures and groups not sharing lowercase letters reflect significant (*P*<0.05) mean differences in response to media osmolality.

The patterns of Prl release from Prl cell incubations by 6 h were more evident and consistent than those observed by 1h (Fig. 2). Prl_188_ released from both SW- and FW-tilapia Prl cells showed thermal, osmotic and interaction effects. In SW fish, a hyposmotic effect was only observed at 32°C, while Prl release increased at 32°C compared with other incubation temperatures in hyposmotic and isosmotic media (Fig. 2A). In FW fish, the thermally-induced Prl_188_ release was observed in cells incubated in all osmotic conditions (Fig. 2B); a five-fold rise in Prl_188_ release was seen in cells incubated in hyposmotic media at 32°C compared with those at 20°C. Hyposmotically-induced Prl_188_ realese, however, was only observed at 26 and 32°C (Fig. 2B). By contrast, an inverse relationship between Prl_177_ release and medium osmolality was seen in both SW and FW fish at all temperatures (Fig. 2C and 2D). In both SW- and FW-acclimated fish, Prl release was increased in hyposmotic media and inhibited by a drop in temperature.

### 2. Effects of temperature and osmolality on prl mRNA expression

**Figure 3:**
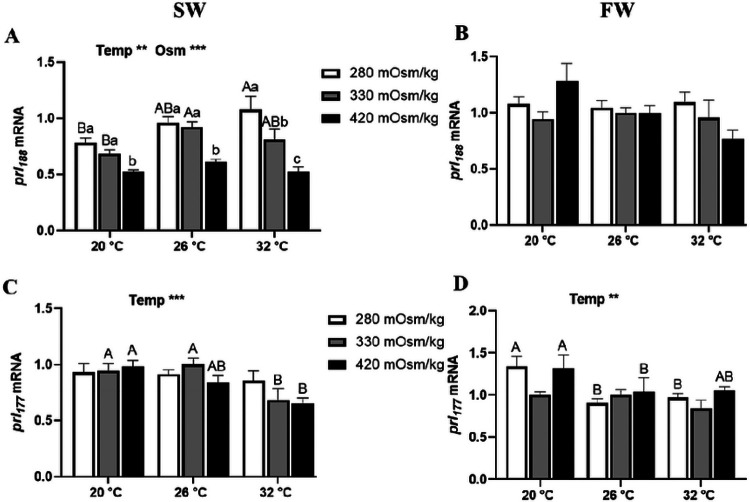
Effects of incubation osmolality and temperature on the mRNA expression of *prl*_*188*_ and *prl*_*177*_ in SW-acclimated tilapia (A and C) and FW-acclimated tilapia (B and D) Prl cells after 6 h of incubation. Data are expressed as mean fold change from the isosmotic and isothermal (330 mOsm/kg:26 °C) group ± SEM (n=6-8). The effects of osmolality and temperature were analyzed by two-way ANOVA (*P<0.05, **P<0.01, ***P<0.001). When there was a significant effect of temperature (Temp), media osmolality (Osm) or interaction (Interact), group comparisons were conducted using protected Fisher’s LSD test. Groups not sharing uppercase letters indicate significant (*P*<0.05) mean differences in response to incubation temperatures and groups not sharing lowercase letters reflect significant (*P*<0.05) mean differences in response to media osmolality.

The mRNA expression of *prl_188_* and *prl_177_* in tilapia Prl cells incubated for 6 h are shown in Fig. 3. In SW-acclimated tilapia, *prl_188_* expression was inversely related to media osmolality at all temperatures, and directly related to temperature in isosmotic and hypoosmotic conditions (Fig. 3A). By contrast, in FW-acclimated fish, *prl_188_* did not vary among treatments (Fig. 3B). In both SW- and FW-acclimated fish, temperature was the only factor affecting *prl_177_* mRNA expression (Fig. 3C and D). In SW-fish, *prl_177_* in isosmotic and hyperosmotic media was higher at 20°C than at 32°C, while in FW-fish, *prl_177_* in hyposmotic and hyperosmotic conditions were higher at 20°C compared with 26°C.

### 3. Effects of temperature and osmolality on prlr mRNA expression

**Figure 4:**
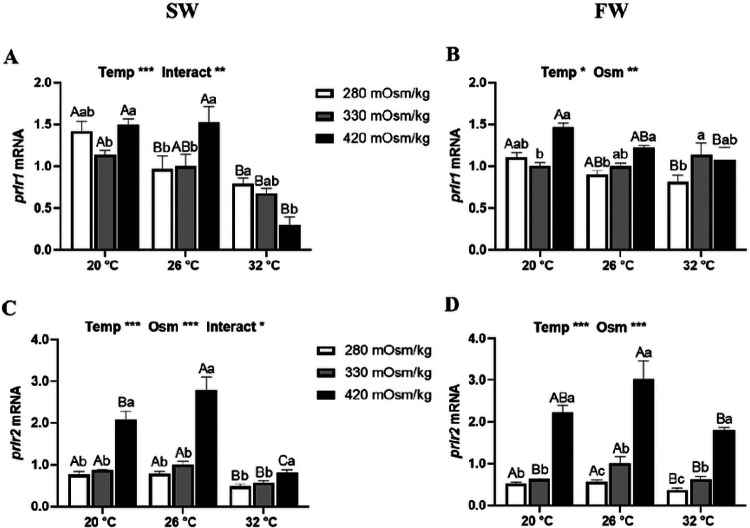
Effects of incubation osmolality and temperature on the mRNA expression of *prlr1*and *prlr2* in SW-acclimated tilapia (A and C) and FW-acclimated tilapia (B and D) Prl cells after 6 h of incubation. Data are expressed as mean fold change from the isosmotic and isothermal (330 mOsm/kg:26 °C) group ± SEM (n=6-8). The effects of osmolality and temperature were analyzed by two-way ANOVA (*P<0.05, **P<0.01, ***P<0.001). When there was a significant effect of temperature (Temp), media osmolality (Osm) or interaction (Interact), group comparisons were conducted using protected Fisher’s LSD test. Groups not sharing uppercase letters indicate significant (*P*<0.05) mean differences in response to incubation temperatures and groups not sharing lowercase letters reflect significant (*P*<0.05) mean differences in response to media osmolality.

Main effects of temperature and osmolality were observed in the transcription of *prlr1* and *prlr2* from Prl cell incubations (Fig. 4). In SW-acclimated tilapia, *prlr1* was downregulated at 32°C compared with other temperatures, while the osmotic effect changed according to temperature (Fig. 4A). In FW-acclimated fish, *prlr1* was upregulated as media osmolality increased at all temperatures, while inversely related with temperature in hypo- and hyperosmotic incubations (Fig. 4B). A notable increase of *prlr2* (up to ~ 3-fold) was observed in Prl cells of both SW- and FW-acclimated fish incubated in hyperosmotic media at all temperatures (Fig. 4C and D). At 32°C, expression of *prlr2* in Prl cells of SW fish was downregulated at all media osmolalities compared with the other incubation temperatures (Fig. 4C).

### 4. Effects of temperature and osmolality on trpv4 mRNA expression

**Figure 5:**
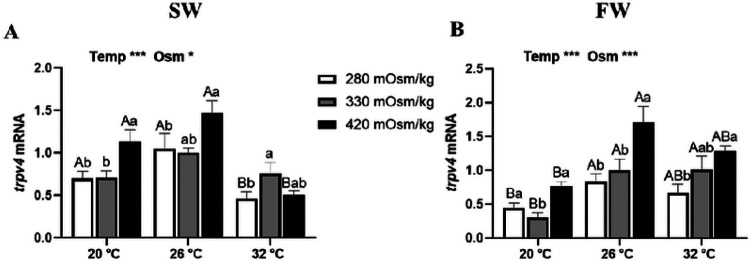
Effects of incubation osmolality and temperature on the mRNA expression of *trpv4*in SW-acclimated tilapia (A) and FW-acclimated tilapia (B) Prl cells after 6 h of incubation. Data are expressed as mean fold change from the isosmotic and isothermal (330 mOsm/kg:26 °C) group ± SEM (n=6-8). The effects of osmolality and temperature were analyzed by two-way ANOVA (*P<0.05, **P<0.01, ***P<0.001). When there was a significant effect of temperature (Temp), media osmolality (Osm) or interaction (Interact), group comparisons were conducted using protected Fisher’s LSD test. Groups not sharing uppercase letters indicate significant (*P*<0.05) mean differences in response to incubation temperatures and groups not sharing lowercase letters reflect significant (*P*<0.05) mean differences in response to media osmolality.

There were main effects of osmolality and temperature in *trpv4* expression in Prl cells of tilapia acclimated to both FW and SW (Fig. 5). In Prl cells of SW-acclimated tilapia, *trpv4* expression was higher in hyperosmotic media, with the exception of incubations carried out at 32°C, where expression was highest in isosmotic conditions (Fig. 5A). In FW-fish, *trpv4* expression was increased by rises in extracellular osmolality (Fig. 5B). The expression of *trpv4* was inhibited in Prl cells incubated at 20°C compared with that at 26°C in fish acclimated to FW, but not those in SW.

### 5. Effects of temperature and osmolality on TF transcript mRNA expression

**Figure 6:**
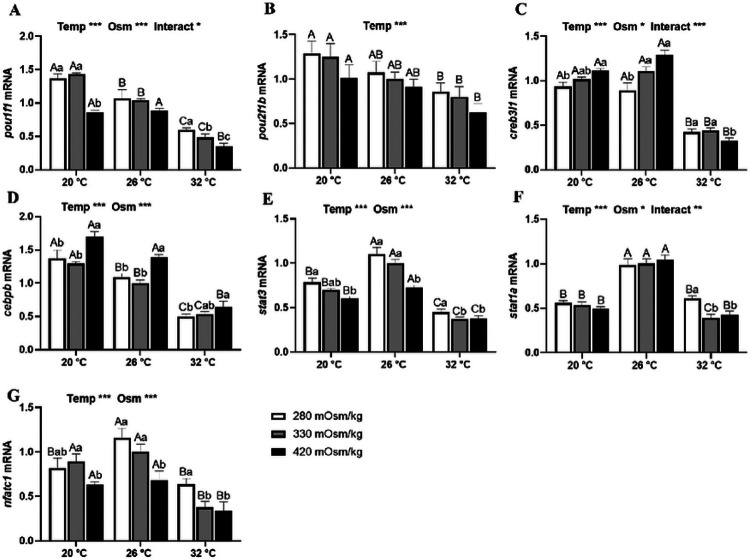
Effects of incubation osmolality and temperature on the mRNA expression of *pou1f1*(A), *pou2f1b* (B), *creb3l1* (C), *cebpb* (D), *stat3* (E), *stat1a* (F) and *nfatc1* (G) in SW-acclimated tilapia Prl cells after 6 h of incubation. Data are expressed as mean fold change from the isosmotic and isothermal (330 mOsm/kg:26 °C) group ± SEM (n=6-8). The effects of osmolality and temperature were analyzed by two-way ANOVA (*P<0.05, **P<0.01, ***P<0.001). When there was a significant effect of temperature (Temp), media osmolality (Osm) or interaction (Interact), group comparisons were conducted using protected Fisher’s LSD test. Groups not sharing uppercase letters indicate significant (*P*<0.05) mean differences in response to incubation temperatures and groups not sharing lowercase letters reflect significant (*P*<0.05) mean differences in response to media osmolality.

**Figure 7:**
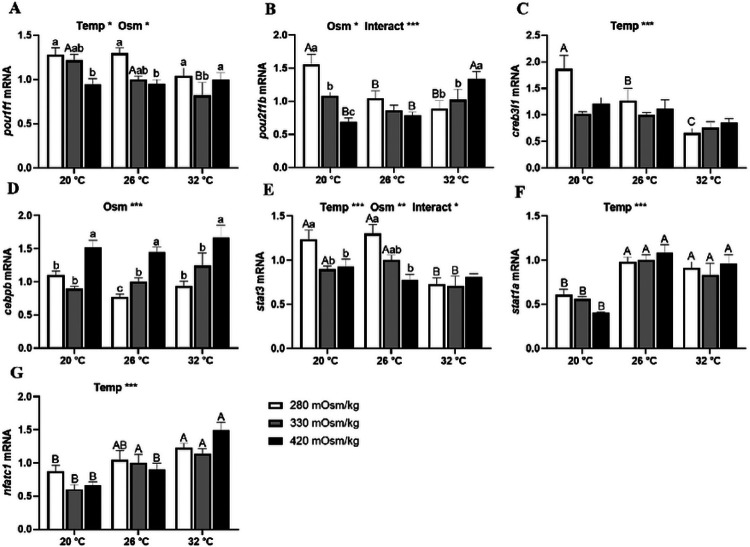
Effects of incubation osmolality and temperature on the mRNA expression of *pou1f1*(A), *pou2f1b* (B), *creb3l1* (C), *cebpb* (D), *stat3* (E), *stat1a* (F) and *nfatc1* (G) in FW-acclimated tilapia Prl cells after 6 h of incubation. Data are expressed as mean fold change from the isosmotic and isothermal (330 mOsm/kg:26 °C) group ± SEM (n=6-8). The effects of osmolality and temperature were analyzed by two-way ANOVA (*P<0.05, **P<0.01, ***P<0.001). When there was a significant effect of temperature (Temp), media osmolality (Osm) or interaction (Interact), group comparisons were conducted using protected Fisher’s LSD test. Groups not sharing uppercase letters indicate significant (*P*<0.05) mean differences in response to incubation temperatures and groups not sharing lowercase letters reflect significant (*P*<0.05) mean differences in response to media osmolality.

Main effects of temperature and osmolality were observed in the mRNA levels of most TF transcripts from Prl cell incubations of tilapia acclimated to SW and FW (Figs. 6 and 7). In SW-acclimated tilapia, expression of both *pou1f1* (Fig. 6A) and *pou2f1b* (Fig. 6B) was inhibited by high temperature. Expression of *pou1f1* was inversely related with media osmolality at both high and low temperatures while there was no osmotic effect on *pou2f1b* expression. Expression of *creb3l1* (Fig. 6C) and *cebpb* (Fig. 6D) was also inhibited by high temperature. Both *creb3l1* and *cebpb* were elevated by hyperosmotic media, except for *creb3l1* expression at 32°C. Both *stat3* (Fig. 6E) and *stat1a* (Fig. 6F) were highly expressed at 26°C; expression in both high and low temperatures was lower than isothermal controls. The expression of *stat3* was inversely related with osmolality at all temperatures; *stat1a* expression was elevated by hyposmotic media only at 32°C. Similarly, *nfatc1* expression was suppressed in hyperosmotic media (Fig. 6G). High temperature inhibited *nfatc1* in isosmotic and hyperosmotic media, while both high and low temperatures suppressed hyposmotically-induced *nfatc1* expression.

In Prl cells of FW-acclimated tilapia, *pou1f1* expression was inversely related with extracellular osmolality at 20°C and 26°C (Fig. 7A). A thermal effect on *pou1f1* was only seen in isosmotic conditions, where it was downregulated at 32°C. In isothermal conditions, *pou2f1b* expression was not affected by extracellular osmolality (Fig. 7B). At 20°C, *pou2f1b* was inversely related to osmolality, however, at 32°C, it increased with osmolality. The expression of *pou2f1b* in hyposmotic media was inhibited by a rise temperature, while its expression in hyperosmotic media was elevated at 32°C. As temperature rose, *creb3l1* was downregulated in hyposmotic media (Fig. 7C). There was no temperature effect on *cebpb* expression; hyperosmotically-induced transcription was observed at all temperatures (Fig. 7D). Hyposmotically-induced *stat3* expression was observed at 20°C and 26°C, while transcripts in hyposmotic and isosmotic conditions were inhibited at 32°C (Fig. 7E). Medium osmolality did not affect *stat1a* expression (Fig. 7F); transcription was inhibited by low temperature at all media osmolalities. Similarly, *nfatc1* was not affected by osmolality, but was inhibited by lower temperatures (Fig. 7G).

## Discussion

Stemming from the recent finding that tilapia Prl cells are thermosensitive ^27^ in addition to their well established role in osmoreception, the present study examined the interactions between osmotic and thermal stimuli in Prl cells of Mozambique tilapia acclimated to either FW or SW. Our findings indicate that: **1**) A rise in temperature increases Prl release *in-vitro* as early as 1 h; **2**) The osmotic-sensitivity of Prl release is lost at 20°C; **3)** Tilapia acclimated to SW are more responsive to changes in temperature than those acclimated to FW; **4)** *prlr2* expression is inversely related with circulating levels of Prl and is inhibited by a rise in temperature; **5**) *trpv4* responded differentially to temperature depending on the acclimation salinity of fish; **6**) Most of the TF transcripts in Prl cells of SW-acclimated tilapia decrease their mRNA levels in response to an elevation in termperature.

Mozambique tilapia Prl cells are osmoreceptors ^7^ which have been recently described to also respond to physiologically relevant increases in temperature by increasing Prl release ^27^. The control of Prl release by environmental salinity, *in vivo*, and extracellular osmolality, *in vitro*, is well studied ^17,19,49,50^. Prl cells from FW-acclimated tilapia release more Prl than their SW-counterparts, and respond more robustly to changes in extracellular osmolality ^17,26^. As expected, robust hyposmotically-induced Prl_188_ release was observed in FW-acclimated tilapia Prl cells, especially by 6 h of incubation; a rise in temperature amplified this effect. In our previous study, both dispersed Prl cells and RPD organoids responded to higher temperatures by elevating Prl_188_ release by 6 h of incubation (24). The present study, however, is the first to test the response of dispersed tilapia Prl cells subjected to 20°C, which interestingly blocked hyposmotically-induced release of Prl_188_, but not Prl_177_. A previous *in-vivo* study showed no changes in plasma Prl_188_ when tilapia were exposed to temperatures ranging between 20°C and 35°C, though plasma cortisol decreased at higher temperatures ^51^. Inasmuch as cortisol has been reported to inhibit Prl release^52-54^,the thermally-induced rise in Prl release observed in the present study is consistent with lower cortisol in circulation. Moreover, because a rise in temperature also increases Prl cell volume, which mediates Prl release ^27^, the observed inhibition of Prl release at 20°C by 6 h may be directly linked to cell volume change.

Inasmuch as Prl is pleiotropic, a rise in temperature might affect several other key functions of Prl, including growth and reproduction. In fact, a previous study has shown that warmer water (32°C) increases growth, while temperatures as low as 22°C resulted in stunted growth ^55^. Moreover, Prl has been linked with testosterone production and gonadal activity of tilapia ^56^ underscoring the linkage between elevated Prl at high temperatures and increased sexual maturity. While the osmotic sensitivity of Prl_188_ release was lost at 20°C, it did not affect the osmotic responsiveness of Prl_177_ release.. Prl_177_ also exerts somatotropic actions in tilapia ^57^ and while a reduction in Prl_177_ at 20°C is consistent with lower growth, the retention of its hyposmotic response at that temperature may be vital for FW acclimation in cooler temperatures. The differential responsiveness of Prl_188_ and Prl_177_ to extracellular osmolality has also been suggested to underlie the observed differences in salinity tolerance between Mozambique tilapia and its congener Nile tilapia, *Oreochromis niloticus*^58^. Based on the thermal modulation of osmotic responses observed in the present study, it would also be tenable that variations in temperature act in concert with changes in salinity in determining the species-specific environmental regulation of Prl in teleosts.

In FW-acclimated tilapia, *prl* mRNA did not show any osmosensitivity, consistent with previous studies ^17,59^ and the notion that *prl* mRNA levels in FW-fish may be at or near the maximum transcriptional activity and therby unresponsive to further osmotic stimulation ^26^. On the other hand, Prl cells from SW-acclimated tilapia contain low levels of Prls, and therefore, activate *prl_177_* and *prl_188_* transcription in hyposmotic conditions ^26,37,59^. Accordingly, we observed *prl_188_* to be responsive to osmotic stimuli in Prl cells of SW-tilapia. Furthermore, in SW-tilapia, *prl_177_* was not as osmotically sensitive as *prl_188_*, consistent with previous observations ^17^. The two *prl* transcripts showed opposite expression patterns in response to thermal stimuli. The expression of *prl_188_* peaked with a rise in temperature in hyposmotic media, indicating that a combination of heat and low osmolality synergizes to maximally induce *prl_188_* transcription. Thermally-induced Prl release was recently shown to be mediated, at least partially, by a cell-volume dependent mechanism, similar to that involved in osmotically induced Prl release ^27^. Consistently, the transcription of *prl_188_* may be activated in similar fashions by thermal and osmotic stimuli, and further augmented in environments that are both hyposmotic and warm.

In Mozambique tilapia, the biological effects of Prls are mediated by Prlr1 and Prlr2, whose transcription in target tissues is also characterized by high osmotic sensitivity ^17,60,61^. Expression of both *prlrs* was affected by temperature and osmolality. Consistent with previous studies ^17,22^, the relationship of *prlr2* was inversely related to extracellular osmolality in Prl cells of both SW- and FW-acclimated tilapia. Both *prlrs* were decreased by incubation at 32°C compared with cooler temperatures (Fig. 4C and 4D). Regardless of the circulating levels of Prls, the environmental control of their receptors are implicated in modulating the hormonal actions ^17,61^. The observed decreases in *prlrs* with a rise in temperature, especially in Prl cells of SW-acclimated fish incubated in hyperosmotic media, suggests that Prl’s effects in high temperature may be attenuated.

The transduction of hyposmotic stimuli in tilapia Prl cells is dependent on the entry of extracellular Ca^2+ 50,62,63^ through trpv4 channels^31,34^. Trpv4 is sensitive to many stimuli including osmotic pressure and heat ^64,65^. We observed an increase in *trpv4* proportional to that of extracellular osmolality, though this relation was attenuated at the highest incubation temperature. The responses of *trpv4* to thermal and osmotic sensitivity differed between Prl cells from FW- and SW-acclimated tilapia, though genereally, the transcript was most highly expressed at 26°C. In our previous study, Prl cells of FW-acclimated tilapia increased *trpv4* in response to an elevation in temperature ^27^. In the present study, this trend was confirmed, but only when comparing cells inclubated at 20°C and 26°C. In SW-acclimated tilapia, however, there were no clear effects of thermal regulation of *trpv4* expression. Acclimation history plays a vital role in *trpv4* expression and it has been reported that Prl cells of SW-acclimated tilapia express four-fold higher *trpv4* than their FW counterparts ^59^. Hence, a decreased in *trpv4* expression observed in Prl cells of SW-acclimated fish incubated at 32°C may indicate the attenuation of cellular sensitivity to extracellular Ca^2+^ entry in response to environmental stimuli, similar to the response of *prlrs*. It is well accepted that strict regulation of Ca^2+^ concentrations in the cytosol is important in Ca^2+^-mediated cell signaling ^66^; a rise in cellular Ca^2+^ concentration beyond optimum levels may lead to cytotoxicity and cellular apoptosis ^67^. Hence the thermally-induced downregulation of *trpv4* in SW-acclimated tilapia could also serve as a protection mechanism to prevent Ca^2+^ toxicity.

The transduction of osmotic stimuli into the activation of *prl* transcription is largely regulated by the activity of TFs and TF modules (TFMs) that operate in the promoter regions of *prl_188_* and *prl_177_* genes ^29,37^. In Prl cells of tilapia acclimated to SW, expression of TF transcripts was more sensitive to both thermal and osmotic stimuli compared with those in FW. Pit1 and Oct1 have been reported to regulate *prl* transcription in fish and mammalian models^38,68,69^. In the tilapia RPD, *pou1f1* and *pou2f1b* were the most highly expressed transcripts of Pit and Oct1, respectively ^29^. Moreover, *pou1f1* expression was inversely related with osmolality in SW-acclimated tilapia ^37^. Both *pou1f1* and *pou2f1b* were inhibited by a rise in temperature. Inhibition of these TFs by high temperature reinforces the notion of a compensatory mechanism that attenuates thermally-induced Prl release. The osmotic sensitivity observed at 32°C indicates that Prl cells are capable of retaining osmoreceptive functions at higher temperatures. In FW-fish, both *pou1f1* and *pou2f1b* were inversely related to osmolality at 20°C. At this low temperature, *Prl_188_* release was minimal and unresponsive to changes in media osmolality; *prl_188_* expression was unresponsive to both osmotic and thermal stimuli by 6 h. Collectively, these results suggest that, in FW-acclimated tilapia, Prl cells maintained their osmosensitivity through *pou1f1* and *pou2f1b* at 20°C even though *prl_188_* mRNA was unchanged across treatments, possibly as result of pre-existing elevated levels of transcripts and stored Prl_188_.

Hyposmotically-induced Prl release has also been shown to involve the cAMP second messenger system ^35,70^. To address downstream changes in this second messenger system, we characterized the response of two transcripts of CREB and CEBP, *creb3l1* and *cebpb*, respectively, which are prevalent in tilapia Prl cells ^29^. Similar to the pattern of expression observed for POU genes, a rise in temperature inhibited *creb3l1* expression in Prl cells of both SW- and FW-acclimated tilapia. In SW fish, *creb3l1* increased in hyperosmotic media at colder temperatures and was attenuated at 32°C. This expression pattern was quite similar to the expression of *trpv4*, suggesting a linkage between Ca^2+^ and cAMP second messenger systems in the integration of thermal and osmotic responses. By contrast, *creb3l1* was not affected by media osmolality in Prl cells of FW-acclimated tilapia; the only effect observed was the downregulation of the transcript with rising temperature in hyposmotic media. Previously, we reported that *trpv4* increased from 26°C to 32°C in isosmotic conditions, but did not affect *prl_188_* or *prl_177_* mRNA in Prl cells of FW-acclimated tilapia ^27^. The downregulation or unresponsiveness of *creb3l1* to a rise in temperature may, therefore, contribute to the maintenance of both *prls* at stable levels at high temperatures. The current results are also consistent with the high expression of *creb3l1* reported in SW-tilapia RPDs ^29^ and the lack of osmotic responsiveness in Prl cells from FW-acclimated fish ^37^. Similarly, *cebpb* followed the expression pattern of *trpv4*, with upregulation directly proportional to a rise in osmolality. Despite the lack of a thermal effect in Prl cells of FW-acclimated tilapia, *cebpb*’s similarity in response patterns to that of *trpv4* during *in vitro* and *in vivo* elevations in extracellular osmolality ^59^ together with it’s role in encoding an intermediate Ca^2+^ binding protein in the cAMP second messenger system ^71,72^, reinforces the notion that thermo- and osmosensitive TFs linked to Ca^2+^ and cAMP signalling act in concert in the environmental regulation of Prl cells.

Following the binding of Prl to its receptors, Stat proteins mediate the activation of the JAK/STAT signaling pathway ^12^. In the present study, *stat3* expression in Prl cells of SW-acclimated tilapia was induced in hyposmotic medium at all temperatures. Tilapia Prl cells have been shown to positively respond to both Prl_188_ and Prl_177_ *in vitro*, in autocrine fashion, and the extracellular osmolality^36^. Inasmuch as these autocrine responses occur through Prlrs and the activation of JAK/STAT, understanding the thermal and osmotic modulation of these TFs shall provide further insight into the environmental regulation of Prl cells. Both *stat3* and sta1a s were inhibited at 20°C and 32°C, indicating that JAK/STAT signaling is optimized at prevailing ambient temperatures (~ 26°C). We observed Prl release and *prlr* expression to have opposite patterns of response to thermal stimuli. Because Prl_188_ present in the medium has shown to increase Prl release even in hyperosmotic conditions ^36^, the rise in media Prl concentration at 32°C, may have triggered the inhibition of *prlr2* and *stat3* expression at the warmer temperature, as a long-term negative feedback response. The thermal response of *stat1a* was similar to that of *stat3*, although the similarity in osmotic sensitivity was only observed at 32°C. These results indicate that the responses of *stat1a* to environmental changes may not be as sensitive as those of *stat3*, and suggest that during downstream signaling it may be largely sensitive to autocrine regulation by Prls. In Prl cells of FW-acclimated tilapia, *stat3* showed similar osmotic sensitivity as their SW counterparts at lower temperatures. At 32°C, however, osmotic responses were abolished or attenuated in a similar manner as observed with *prlr2*, suggesting that this receptor isoform and *stat3* may be linked during the downstream activation of autocrine signaling. Stat1 is activated by heat in mammalian cell models ^73,74^, though downstream signaling effects may differ if Stat1 dimerizes or binds with Stat3 ^75^. Earlier we found *stat3* levels to be similar in RPDs of SW- and FW-acclimated tilapia but *stat1a* levels to be higher in SW fish ^29^. Therefore, the nuances we observe in *stat* transcripton may be tied with acclimation salinity.

Finally, NFATs have been reported to be activated following rapid Ca^2+^ influx ^76^ and in response to hyperosmotic stress in mammalian cell models and in gills of Atlantic salmon, *Salmo salar*^44,77,78^. Also, NFAT is reported to form TFMs with AP1, a TF that is sensitive to both hypo- and hyperosmotic stress ^76,79-81^. Recently, we reported that the TFM, NFAT_AP1F is activated by both hypo- and hyperosmotic stimuli in tilapia Prl cells^37^. In the present study, *nfatc1* expression was reduced in Prl cells of SW-acclimated tilapia by hyperosmotic conditions. The induction of *nfatc1* at lower media osmolalities may occur, therefore, in response to hyposmotically-induced Ca^2+^ entry. Furthermore, *nfatc1* transcription was attenuated by heat. At 32°C, *trpv4* was also inhibited, suggesting that the attenuation of *nfatc1* could be linked to a reduction in Ca^2+^ influx. At 32°C, similar patterns of transcription were observed in *trpv4*, *creb3l1*, *cebpb* and *nfatc1*, underscoring the importance of free Ca^2+^ entry to activate *prl* transcription. In FW fish, *nfatc1* expression was reduced at 20°C and unresponsive to osmotic stimuli. Similarly, *trpv4* expression was lower at 20°C compared with other incubation temperatures. Together, these results are consistent with the notion that extracellular Ca^2+^ entry into the intracellular space is important to upregulate *nfatc1*.

This study unveils the transcriptional responses to temperature and salinity of molecular regulators involved in *prl* transcription and Prl release in a euryhaline and eurythermal fish model that is highly adaptable to environmental fluctuations. Even though teleosts are considered ectotherms, these results provide evidence of cellular mechanisms of a pleiotropic endocrine system that sense and respond to both thermal and osmotic stimuli. Rises in temperature further augmented hyposmotically-induced Prl release while at the same time attenuating the transcription of TFs and *prlr*s involved in the osmoreceptive and autocrine responses of Prl cells, indicating thereby that both temperature and extracelluar osmolality modulate Prl cell responses in concert. As a result, multiple physiological processes such as growth, development, reproduction and osmoregulation are likely modified following the integrated adaptive responses to changes in environmental temperature and salinity. In light of the predicted increase in frequencies of extreme whether events leading to rising sea surface temperatures and fluctuating salinities ^82,83^, these findings provide insight on how fish may be capable of integrating and responding to these various environmental cues simultaneously.

## Materials And Methods

### Animals

Mature Mozambique tilapia (*O. mossambicus*) of mixed sexes and sizes (200–1200 g) were obtained from stocks maintained at the Hawai’i Institute of Marine Biology, University of Hawai’i (Kaneohe, HI) and at Mari’s Garden (Mililani, HI). Fish were reared in outdoor tanks with a continuous flow of FW or SW under natural photoperiod and fed to satiety once a day with trout chow pellets (Skretting, Tooele, UT). Fish were anesthesized with 2-phenoxyethanol (0.3 ml/L, Sigma Aldrich, St. Louis, MO) and euthanized by rapid decapitation prior to sampling. All methods were carried out in accordance with relevant guidelines and regulations. All experimental procedures and methods were conducted in accordance with the ARRIVE guidelines and approved by the Institutional Animal Care and Use Committee, University of Hawai’i.

### Experiment 1: Effects of temperature on osmotic sensitivity of FW-acclimated tilapia Prl cells

The effects of environmental temperature on the osmotic sensitivity of FW-acclimated tilapia Prl cells were determined *in-vitro* by incubating Prl cells at different combinations of media osmolality and temperature. Thirty FW-acclimated Mozambique tilapia of mixed sex weighing 250-1,150 g were used. Following euthanasia, RPDs of *O. mossambicus* were dissected from the pituitary gland and dispersed Prl cells were prepared as previously described ^30,36^. Briefly, RPDs were treated with 0.125% (wt/ vol) trypsin (Sigma-Aldrich) dissolved in PBS and placed on a gyratory platform set at 120 rpm for 25 min to allow for complete cell dissociation. The cells were centrifuged for 5 min at 1,200 rpm and the supernatant decanted and discarded; cells were resuspended and triturated in trypsin inhibitor (0.125% wt/ vol; Sigma-Aldrich) to terminate the trypsin treatment. Cells were washed with PBS (330 mOsm/kg) twice and then resuspended in isosmotic medium (330 mOsm/kg). The incubation media contained 120 mM NaCl, 4 mM KCl, 0.81 mM MgSO_4_, 0.99 mM MgCl_2_, 2 mM NaHCO_3_, 0.44 mM KH_2_PO_4_, 1.34 mM Na_2_HPO_4_, 2.1 mM CaCl_2_, 10 mM HEPES, 2.77 mM glucose, 2 mM glutamine, 100 IU/mL penicillin, 76.3 IU/mL streptomycin and milli-Q water. A hemocytometer and the trypan blue exclusion test were used to detect cell yield and viability, respectively.

Dispersed Prl cells were preincubated in 300 μL of isosmotic media (200,000 cells/well; 8 replicates per treatment; three plates) at 26°C for 1 h. Then, the cells were rinsed with incubation media (280, 330 or 420 mOsm/kg) twice and incubated under saturated humidity for 6 h. Three culture plates were incubated at three experimental temperatures, 20, 26 and 32°C. After 1 h of incubation and at the end of the incubation, 10 μL of media were collected, diluted 20 times with RIA buffer (0.01 M PBS containing 1% [wt/vol] BSA and 0.1% [vol/ vol] Triton X-100) and stored at −80°C for further analysis. At the end of the incubation, media was removed and, 750 μL of TRI Reagent (MRC, Cincinnati, OH) was added to each well followed by gently mixing with a pipette to detach cells from the bottom for 5 min. The cells and TRI Reagent were then transferred to 1.5 mL tubes and stored at −80°C until further analysis.

### Experiment 2: Effects of temperature on osmotic sensitivity of SW-acclimated tilapia Prl cells

Another *in-vitro* Prl cell incubation experiment was conducted to determine the effects of environmental temperature on the osmotic sensitivity of SW-acclimated tilapia Prl cells by incubating them at different osmolality and temperature combinations. Forty SW-acclimated Mozambique tilapia of mixed sex weighing 200–750 g were used. Following euthanasia, RPDs of *O. mossambicus* were dissected from the pituitary gland, dispersed and loaded into well plates following the same procedure as described for Experiment 1 (200,000 cells/well; 8 replicates per treatment; three plates). Experimental conditions were identical to those employed in Experiment 1.

### Radioimmunoassay

Prl_188_ and Prl_177_ levels in the collected media samples were measured by homologous radioimmunoassay (RIA) using the primary antibodies developed in rabbit against Prl_188_ and Prl_177_ (anti-Prl_188_ and anti-Prl_177_) and secondary antibody raised in goat against rabbit IgG (anti-rabbit IgG) as previously described and validated ^36,84,85^. Dilutions employed for anti-Prl_188_, anti-Prl_177_ and anti-rabbit IgG were 1:35,000, 1:8,000 and 1:100 respectively. Data are expressed as mean fold-change ± SEM (*n* = 8) from the percent release from the isosmotic-isothermal treatment (330 mOsm/kg at 26°C).

### Quantitative real-time PCR (qRT-PCR)

Total RNA was extracted from Prl cells frozen in TRI Reagent following the manufacturer’s protocol and reverse transcribed using a High Capacity cDNA Reverse Transcription Kit (Thermo Fisher Scientific, Waltham, MA). The levels of reference and target genes were determined by the relative quantification method in which relative expression levels are obtained based on a standard curve produced by the amplification of target gene at a range of concentrations using a StepOnePlus real-time qPCR system (Thermo Fisher Scientific). The qPCR reaction mix (15 μL) contained Power SYBR Green PCR Master Mix (Thermo Fisher Scientific), 200 nmol/L forward and reverse primers and 1 μL of cDNA. PCR cycling parameters were as follows: 2 min at 50°C, 10 min at 95°C followed by 40 cycles at 95°C for 15 sec and 60°C for 1 min. Primer sequences are listed in Table 1. The geometric mean of three reference genes (*ef1-α, 18S*, and *β-actin*) was used to normalize target genes. Data are expressed as mean fold-change ± SEM (*n* = 8) from the isosmotic-isothermal treatment (330 mOsm/kg at 26°C).

### Statistics

Data from static incubations of Prl cells were analyzed by two-way ANOVA with osmolality and temperature as main effects. Significant effects of medium osmolality and temperature were followed up by protected Fisher’s LSD test. When necessary, data were log-transformed to satisfy normality and homogeneity of variance requirements prior to statistical analysis. All statistics were performed using Prism 9 (GraphPad, La Jolla, CA) and data are reported as means ± SEM.

## Figures and Tables

**Table 1:** Gene specific primers used for qPCR

Gene	Primer sequence (5’-3’)	R^2^	Efficiency %	Accession number	Reference
*18s*	F: GCTACCACATCCAAGGAAGGC R: TTCGTCACTACCTCCCCGAGT	0.993	70.717	AF497908	25
*ef1a*	F: AGCAAGTACTACGTGACCATCATTG R: AGTCAGCCTGGGAGGTACCA	0.992	74.954	AB075952	86
*β-actin*	F: CTCTTCCAGCCTTCCTTCCT R: ACAGGTCCTTACGGATGTCG	0.987	70.992	FN673689	87
*pou1f1*	F: GGCAATGCTCTCAGCAACAC R: GCATCTCCTGTGCTGCCAT	0.995	77.372	XM_019352661.2	29
*stat3*	F: TATCTGCGTTACCCCGTGTC R: TTTGTGCCTGGGAATCCGTT	0.985	84.833	XM_013269621.3	29
*creb3l1*	F: CAGTTTAACAGCGGAGAAACTCTA R: GGTCACCTGAGAAAGGCACATT	0.998	81.644	XM_005460642.4	29
*stat1a*	F: ACCATCAGAGGCTGCTGAAC R: CAGCCTGGACGGATGAACTT	0.983	89.756	XM_005452305.4	29
*pou2f1b*	F: GGGGACAGATTGCTGGAGTA R: AGCTTCAGCCAAGTCATCGT	0.997	82.643	XM_025903751.1	37
*cebpb*	F: CACATTCACACACCGGAGAC R: CCTGTGAAGCGTACCGTTTT	0.992	91.534	XM_003438913.5	37
*nfatc1*	F: GCCGCTGTAGCTTTAAGTGG R: ACACTGAGGCGAGCTCAAAT	0.997	96.122	XM_003447265.5	37
*prl*_*177*_	F: TGGTTTGGCTCTTTTAACACAGTG R: AGACAATGAGGAGTCACAGAGATTTTAC	0.998	92.719	M27011	25
*prl*_*188*_	F: GGCCACTCCCCATGTTTAAA R: GGCATAATCCCAGGAGGAGAC	0.998	95.252	X93280	25
*prlr1*	F: TGGGTCAGCTACAACATCACTGT R: GGATGGGGCTTGACAATGTAGA	0.983	71.697	EU999785	88
*prlr2*	F: GCCCTTGGGAATACATCTTCAG R: GTGCATAGGGCTTCACAATGTC	0.993	87.509	EU999783	86
*trpv4*	F: AGTGGAGCCCATCAATGAG R: TGTGGTATGTGGGTATGGAG	0.983	90.702	AB648937	34

## Data Availability

Data will be made available on request to the corresponding author, Dr. Andre Seale (seale@hawaii.edu).
